# Supplementary material for: Alternative TSSs are co‐regulated in single cells in the mouse brain
Source: Mol Syst Biol. 2017 May 11;13(5):930. doi: 10.15252/msb.20167374 (PMC5448164; doi:10.15252/msb.20167374)
Supplement: Supplementary file 1 — Appendix [file MSB-13-930-s001.pdf]

List of Figures

Appendix Figure S1: Full gene expression for example genes
Appendix Figure S2: Distribution of molecules around coding start and end sites
Appendix Figure S3: Correlation between genes or major TSS in single cells
Appendix Figure S4: Example of higher correlation using TSS instead of gene count
Appendix Figure S5: Fraction major TSS expression
Appendix Figure S6: Correlation versus Expression for 4 more cell-types
Appendix Figure S7: TSS expression of example genes
Appendix Figure S8: Major TSS consistently higher expressed
Appendix Figure S9: Correlation versus Expression upstream and downstream
Appendix Figure S10: Examples of correlated expression from two alternative TSS in single cells
Appendix Figure S11: Correlation positive after normalization
Appendix Figure S12: Percentage of cells that deviates from expected major to minor TSS ratio
Appendix Figure S13: Correlation versus expression for CA1 neurons with 3′UTR cage peaks
Appendix Figure S14: Distinct peaks of expression within TSSs
Appendix Figure S15: Example of genes with two TSS full length gene expression
Appendix Figure S16: Cst3 expression across cell types

Appendix Figure S1 - Full gene expression for example genes

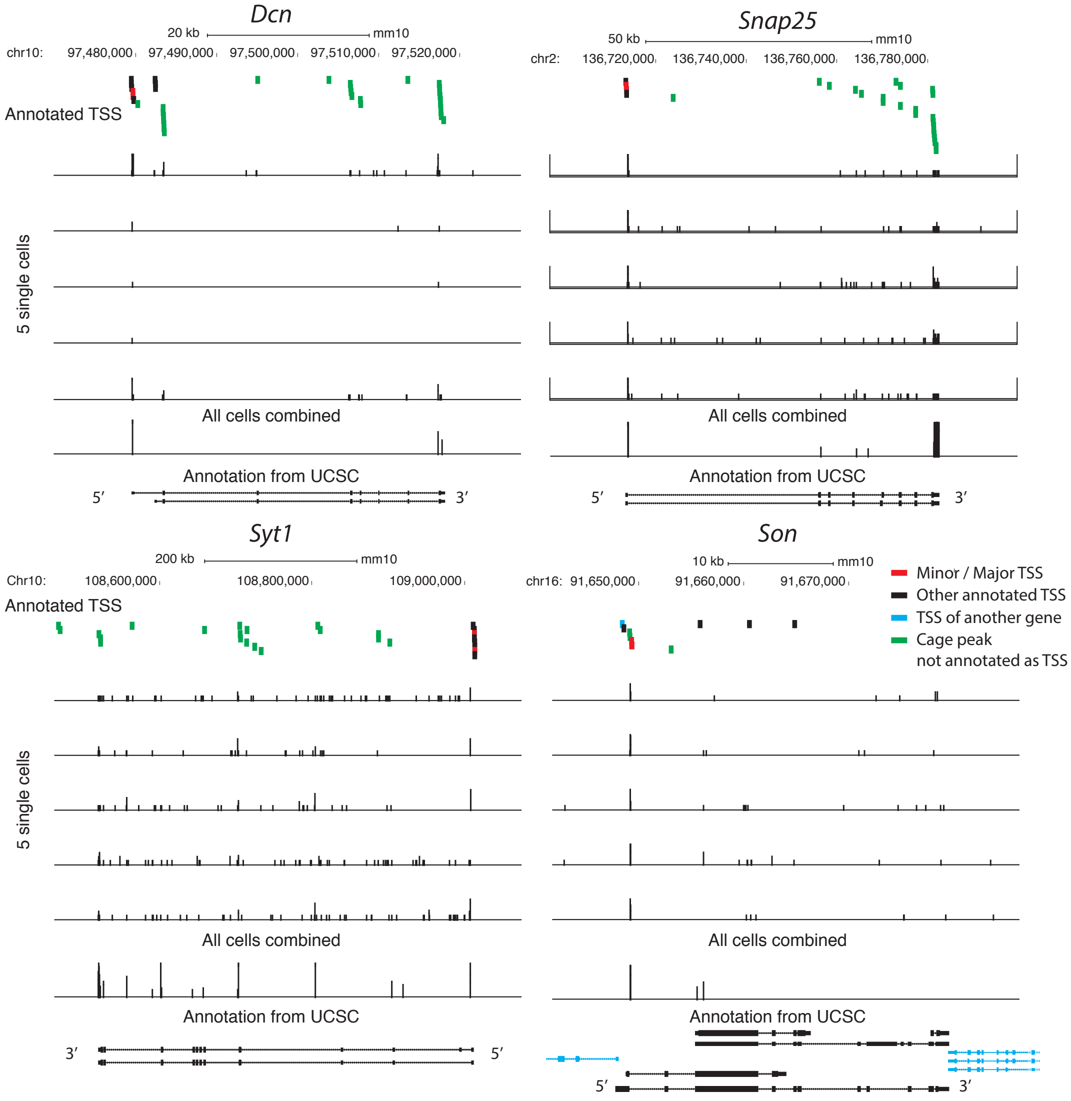

Expression of example genes across the full annotated transcript from UCSC browser for CA1 neurons. Expression is shown as bars where the Y-axis for single cells has a limit of 5 molecules and for all cells combined has a limit of 500 molecules. Major and minor TSS are marked in red, while other CAGE peaks associated with a gene are marked in black. CAGE peaks not associated with a gene are marked in green and TSS from other genes than the gene in focus are marked in blue. Annotations of genes other than the gene in focus are also marked blue. Cells were chosen based on high total TSS expression.

Appendix Figure S2 - Distribution of molecules around coding start and end sites

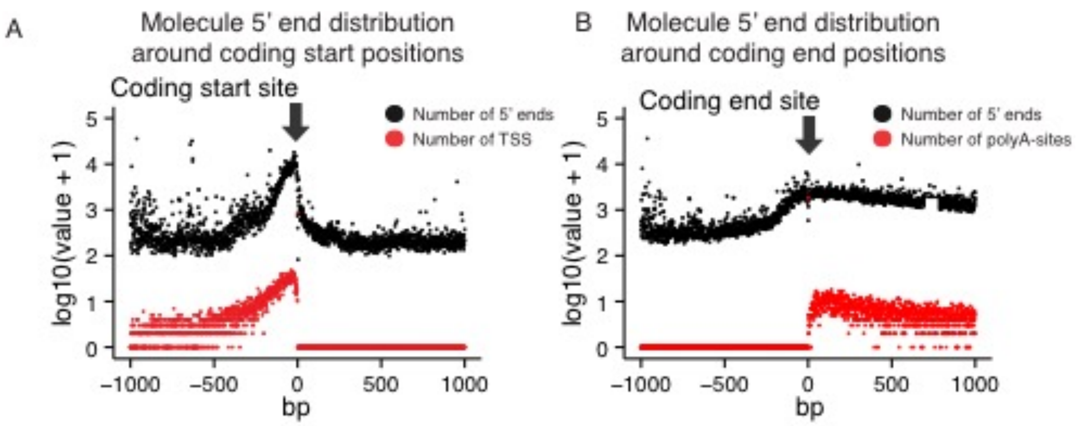

A and B| X-axis shows distribution in base pairs around (A) coding start positions and (B) coding end position. Y-axis shows total CA1-neuron molecules in black and the number of (A) TSS starting or (B) TTS ending in red. The coding start and end positions were taken from RefSeq genes downloaded from UCSC table browser.

Appendix Figure S3 – Correlation between genes or major TSS in single cells

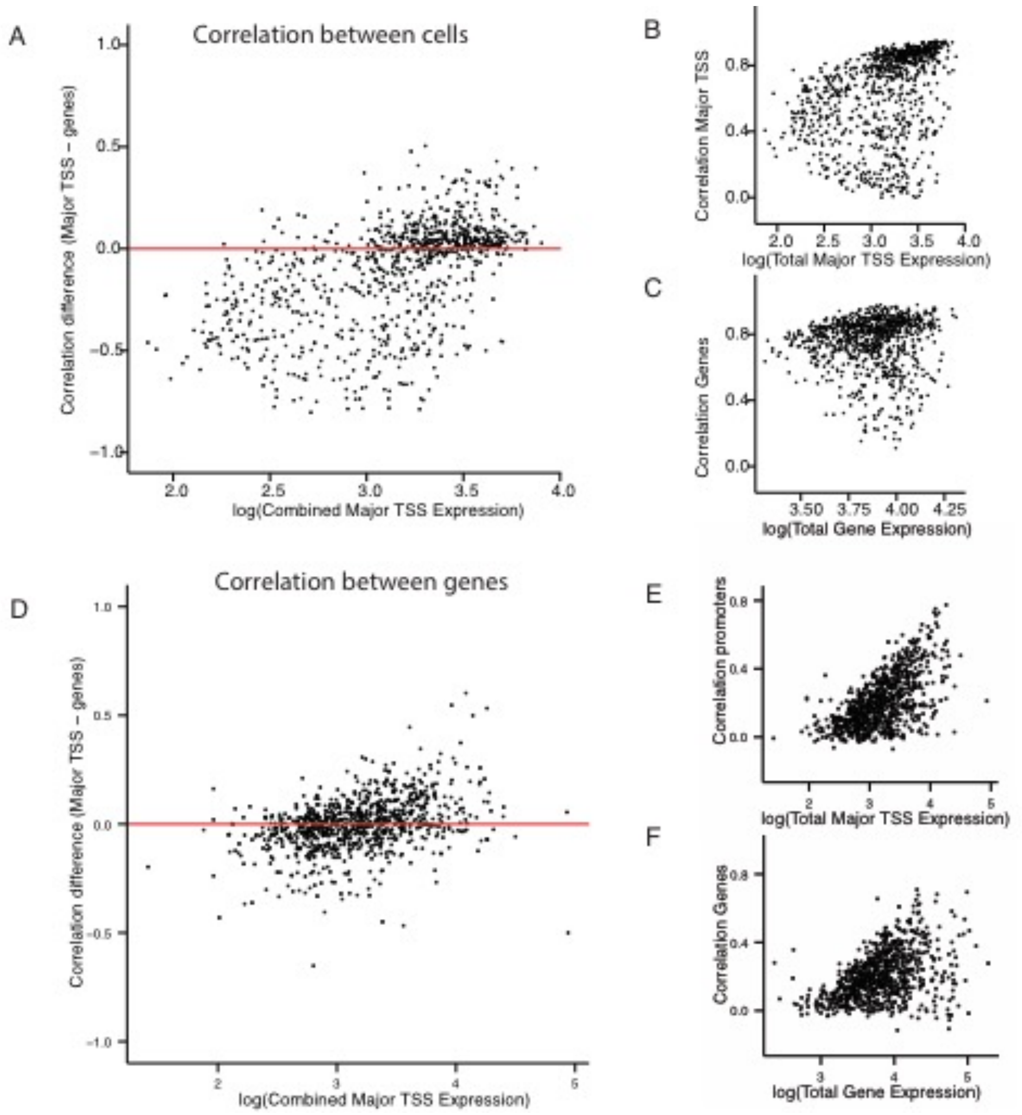

A | Difference in Pearson correlation between total gene and major TSS expression for all genes of the same cell for CA1 neurons. X-axis shows total TSS expression for the two cells being compared, gene expression is not shown. Each dot represents the correlation between two cells and a total of 872 such correlations were made. Only genes with two valid TSS and where annotation existed in both FANTOM5 and RefSeq were included (n=873) when doing the correlations. The average number of molecules mapping to the included RefSeq genes were 8000 and for the two TSS combined 2000.

B | Pearson correlation for single cells using major TSS expression in relation to the combined major TSS expression for the two cells being compared. Each dot is a correlation between two cells as in 3A.

C | Pearson correlation for single cells using genes expression in relation to combined gene expression for the two cells being compared. Each dot is a correlation between two cells as in 3A.

D | Same as supplemental figure 4A, but instead of comparing correlations between cells across genes, correlations are calculated comparing two genes across all cells. Genes are chosen pairwise from the list of genes with valid TSS pairs (in supplemental table 2)

E | Same as 3B but comparing between pairs of genes across all cells

F | Same as 3C but comparing between pairs of genes across all cells

Appendix Figure S4: Example of higher correlation using TSS instead of gene count

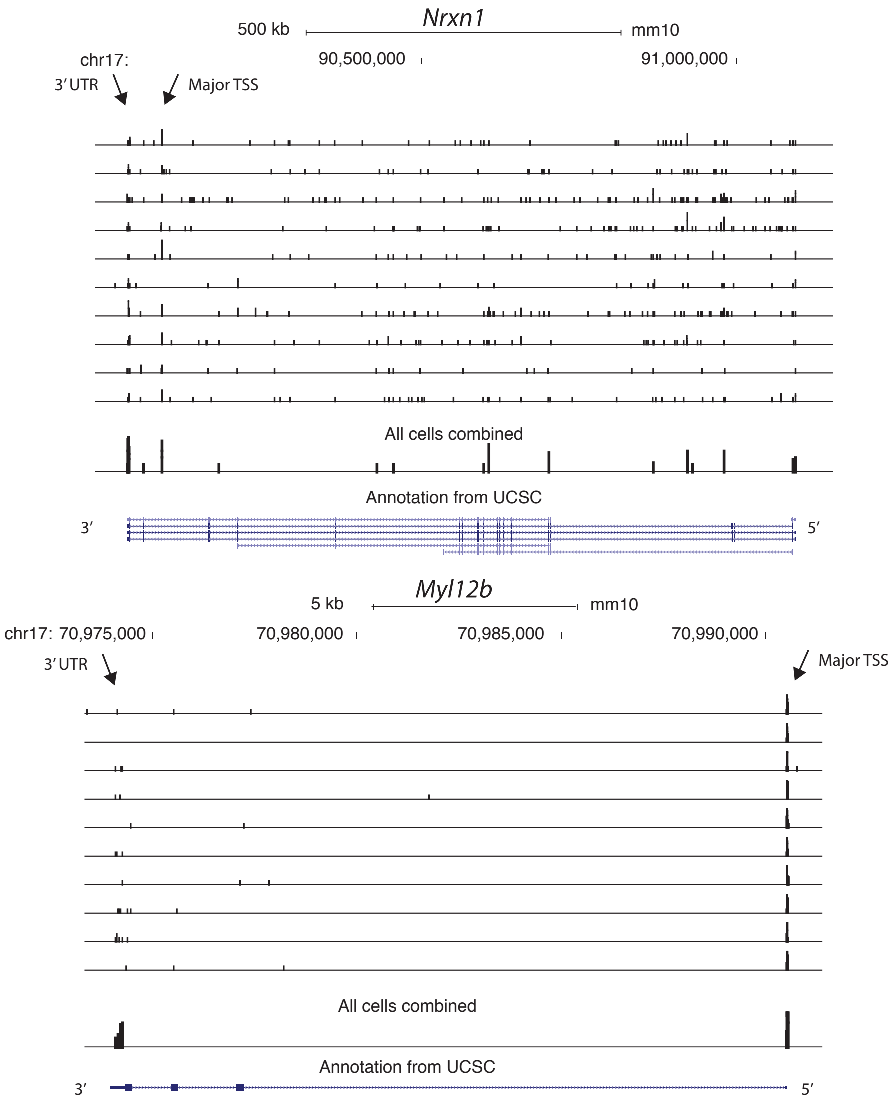

Shows two genes, the genes have a higher correlation if the expression value used is based on read counts from the major TSS (r=0.49), than if the expression value is based read counts from the full gene (r = 0.12). Expression is shown as bars where the Y-axis for single cells has a limit of 5 molecules and for all cells combined has a limit of 500 molecules.

Appendix Figure S5: Fraction major TSS expression

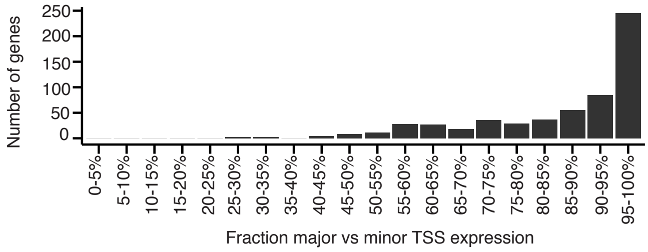

Fraction major versus minor TSS expression for 585 genes expressing > 1 molecules per cell in average in CA1 neurons. X-axis shows the fraction of major TSS expression. Y-axis shows the number of genes with a certain fraction of major TSS expression.

Appendix Figure S6: Correlation versus Expression for 4 different cell-types

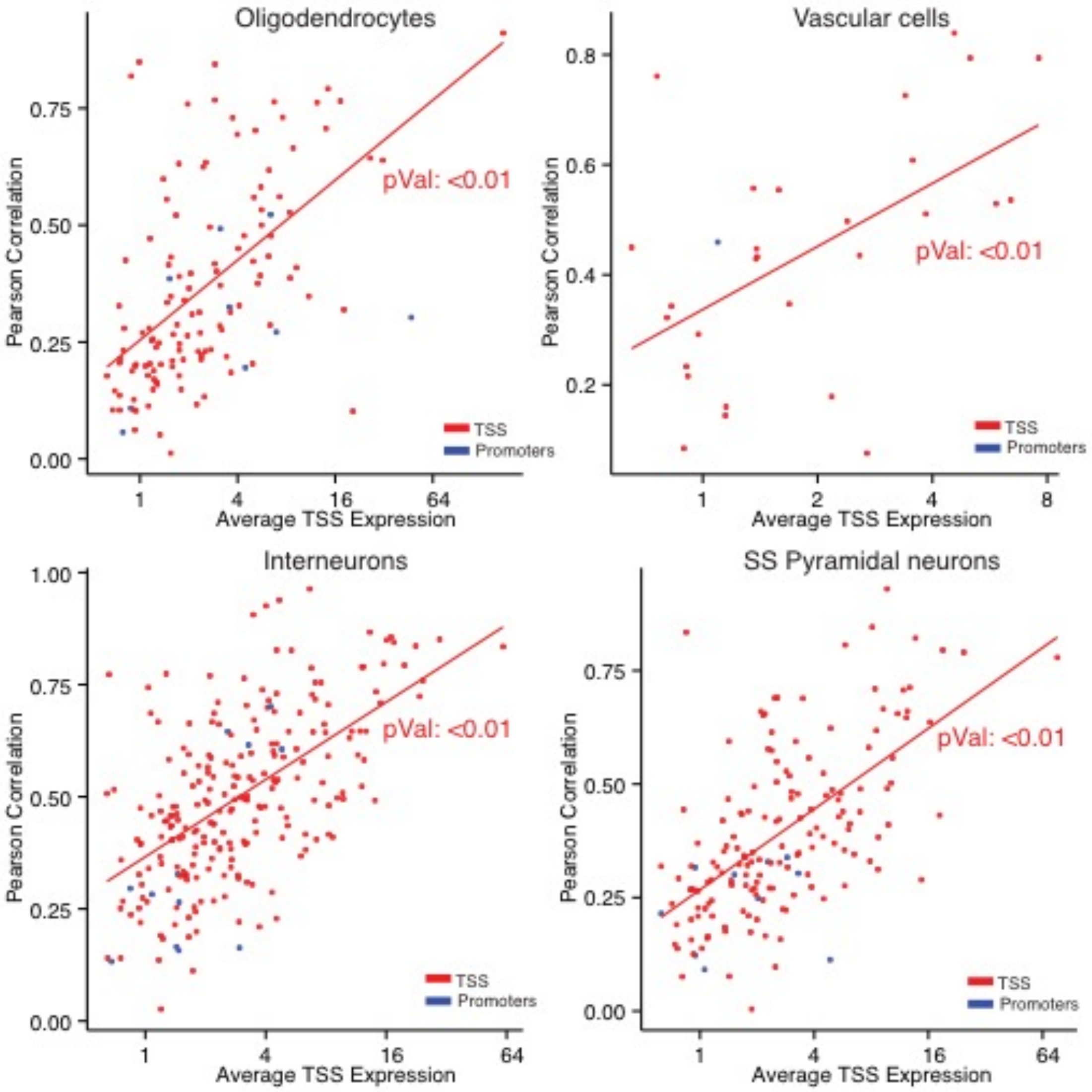

Pearson correlation and expression for four different cell types. Each dot represents a gene and only genes expressing an average of > 0.3 molecules per cell are shown. The colors represent TSS in red and Promoters in blue. Fitted lines for TSSs are drawn and the p-value shows how reliable the fit is.

Appendix Figure S7: TSS expression for example genes

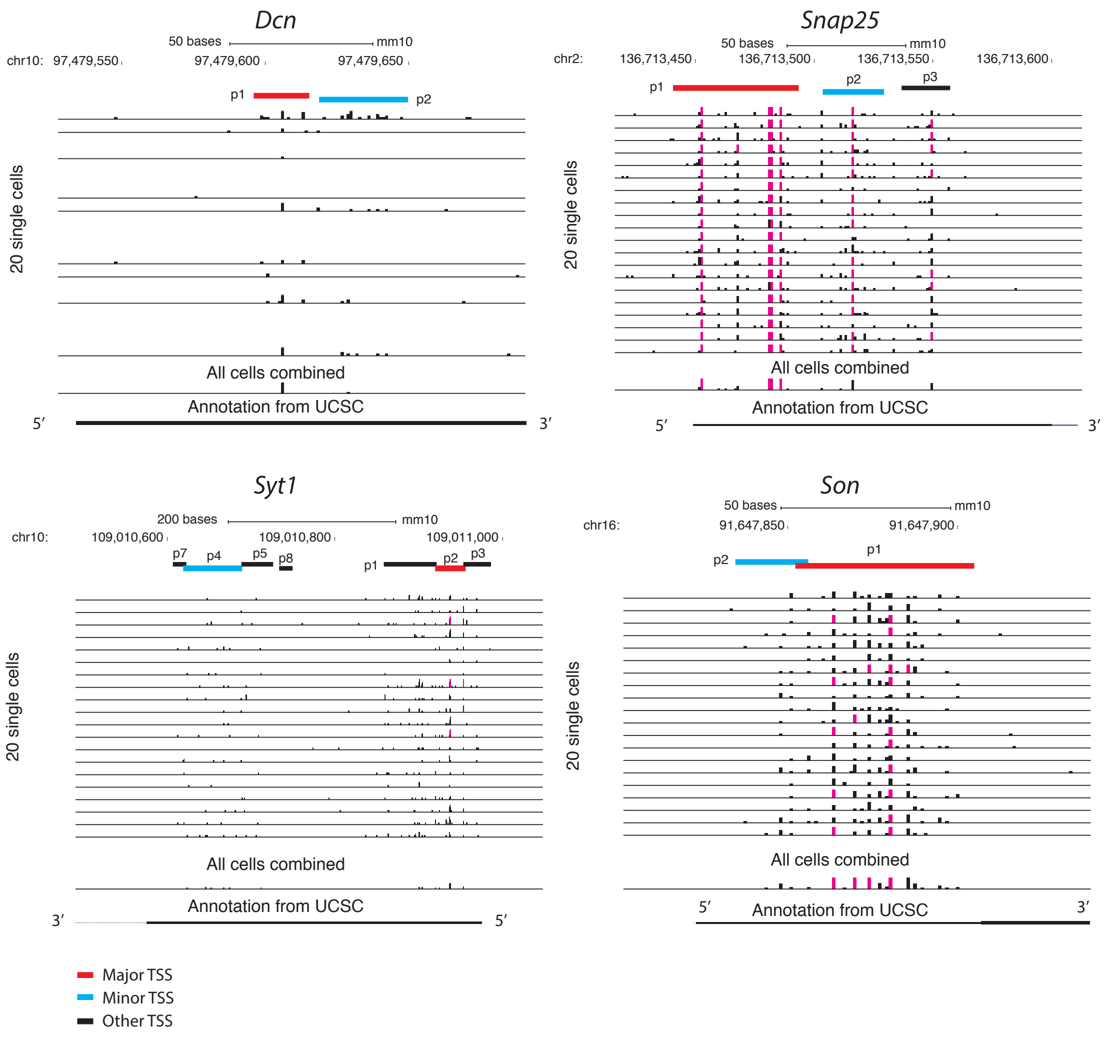

Same figure as supplemental figure 1, but zoomed in on the TSS. The major TSS is colored red, the minor blue and other TSS are colored black. Expression is shown as bars where the Y-axis for single cells has a limit of 5 molecules and for all cells combined has a limit of 1000 molecules. Pink bars shows expression higher than the limit. Note that some cells don't express *Dcn*.

Appendix Figure S8: Major TSS consistently higher expressed

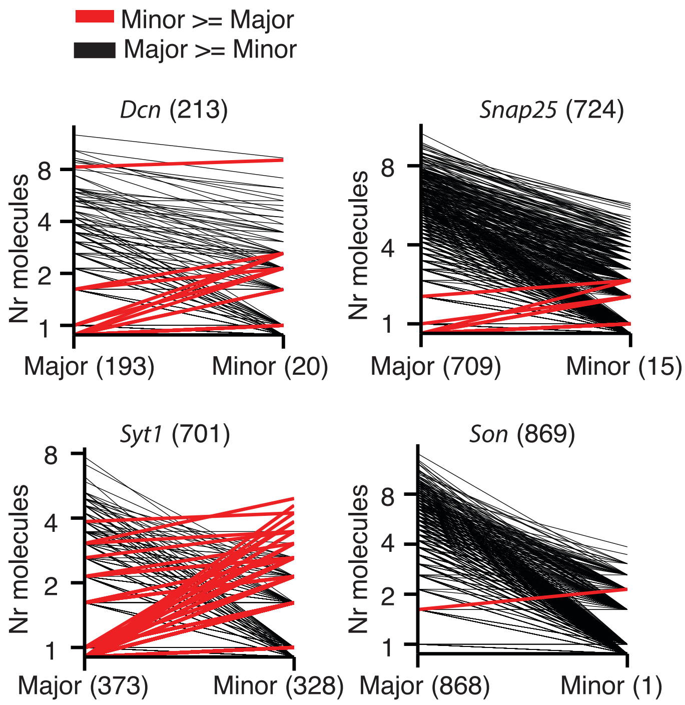

Examples of TSS expression in single CA1 neuron cells. Plots show the number of mRNA molecules detected from the major and minor TSSs in single cells presented to highlight instances of minor TSS preference. Each line connects the read count of the major to the log-read count of the minor TSS of a single cell. Black, major TSS was higher. Red, minor TSS was higher. The number next to the gene symbol shows number of cells where the total expression for the TSSs was greater than zero. The number next to "Major" shows number of cells where cells the major TSS had higher expression than the minor, and the number next to "Minor" shows number of cells where the minor TSS had higher expression. Note that each line can contain more than one cell if two cells have the same expression of the major and minor TSS and that red lines are three times thicker than black lines.

Appendix Figure S9: Correlation versus Expression upstream and downstream

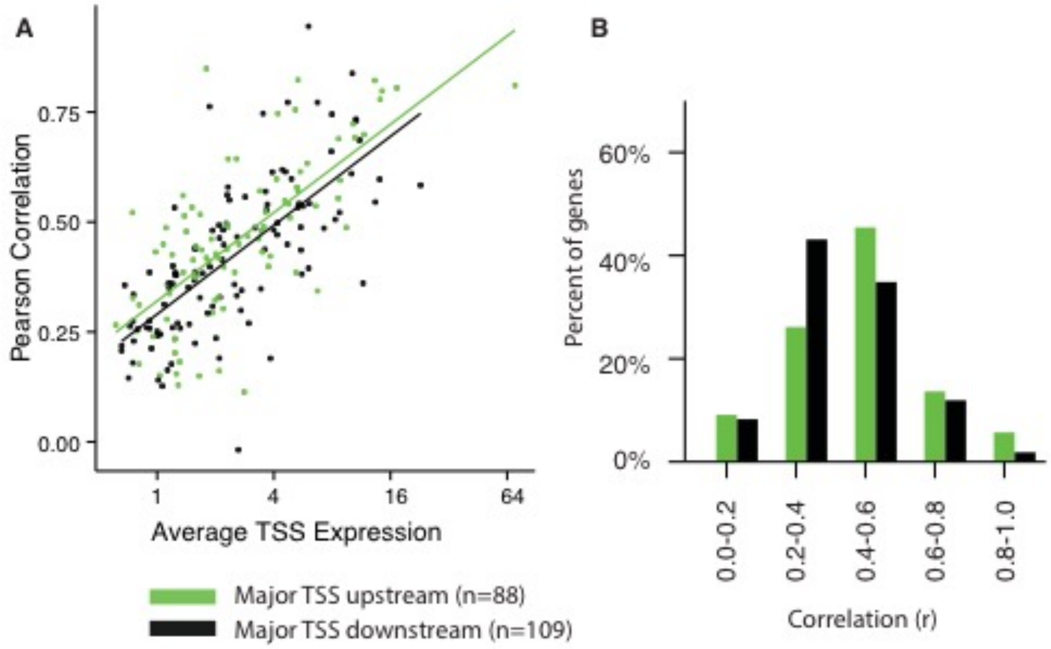

A| Scatterplot showing total expression (horizontal axis) and major/minor TSS correlation coefficient (vertical). Each dot is a gene. Colors represent if the major TSS is upstream (green) or downstream (black) of the minor TSS.

B| Distribution of correlation values where the major TSS is either upstream (green) or downstream (black) of the minor TSS. Histogram based on 197 genes with an expression of at least 0.3 molecules per cell per TSS for CA1 neurons.

Appendix Figure S10: Examples of correlated expression from two alternative TSS in single cells

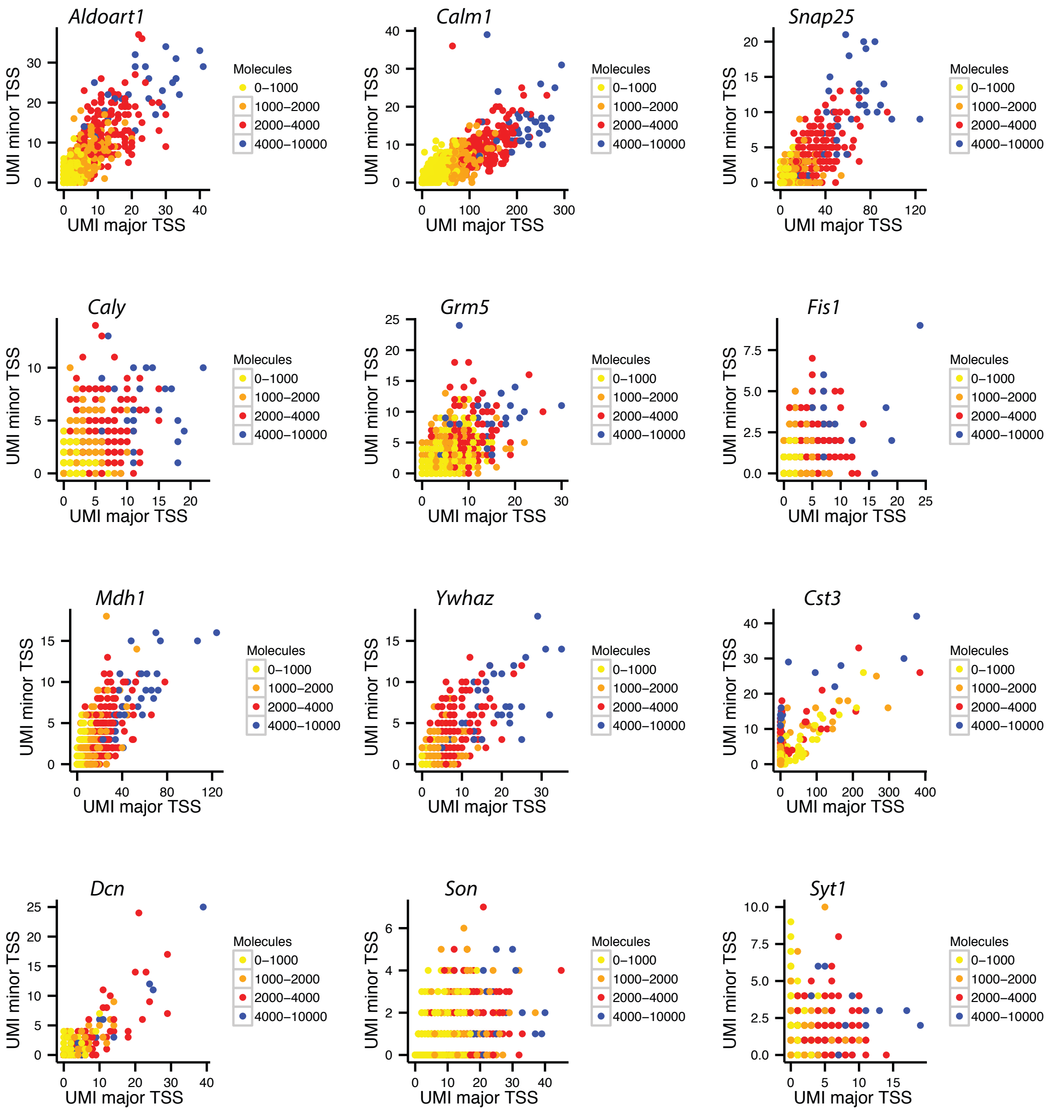

Scatterplot showing molecule count for the major and minor TSS. Each dot represents one cell. 12 highly expressed genes are shown. Cells are colored according to their total TSS expression.

Appendix Figure S11: Correlation positive after normalization

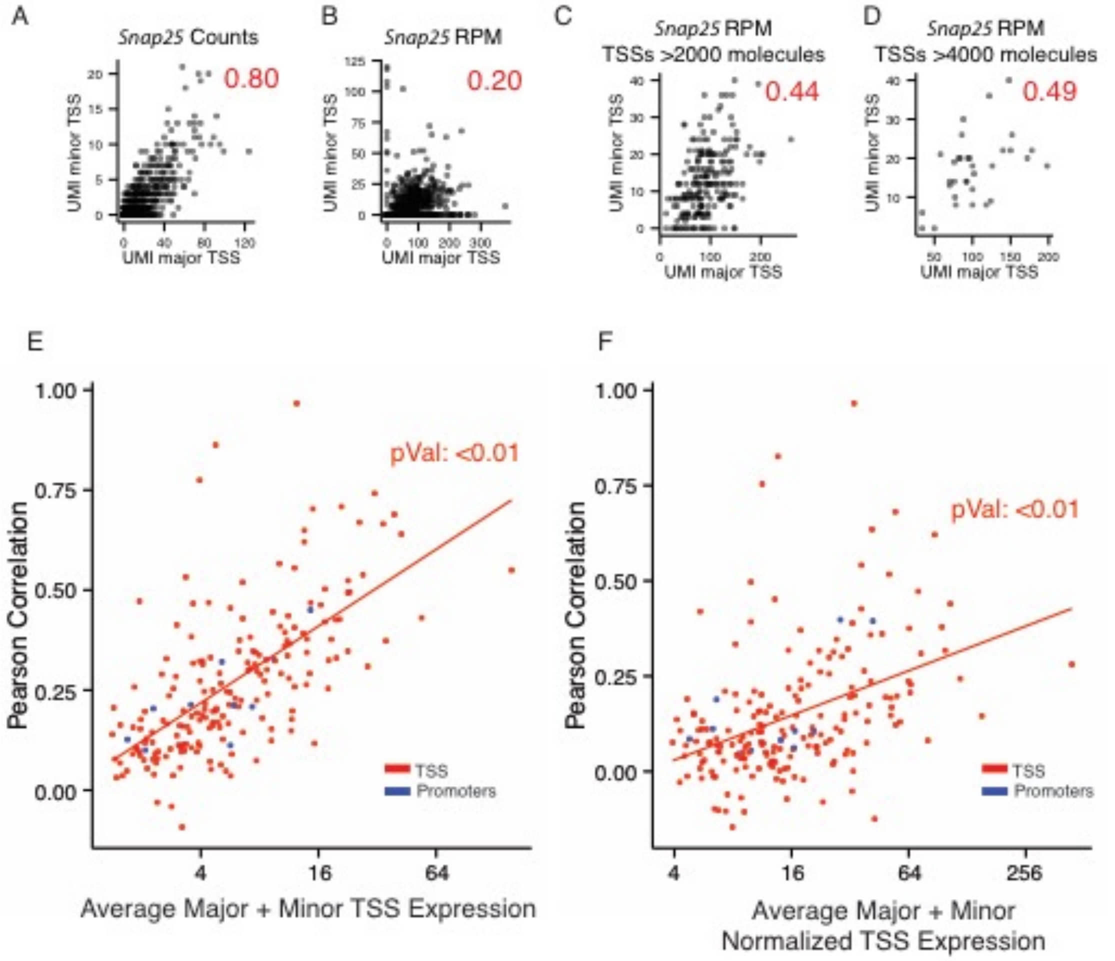

A-D| Major and minor TSS expression for *Snap25* is shown using (A) counts, (B) rpm, (C), rpm but only including cells expressing >2000 molecules and (D) rpm but only including cells expressing >4000 molecules.

E| Scatterplot showing total expression (horizontal axis) and major/minor TSS correlation coefficient (vertical). Each dot is a gene. Only cells expressing >2000 TSS molecules (215 cells) are included. Note that the correlation is lower than in 2F due to that fewer cells are used in the correlation calculation.

F| Scatterplot showing total expression (horizontal axis) and major/minor TSS correlation coefficient (vertical). Each dot is a gene. Only cells expressing >2000 TSS molecules (215 cells) are included and rpm is used instead of read counts.

Appendix Figure S12: Percentage of cells that deviates from expected major to minor TSS ratio

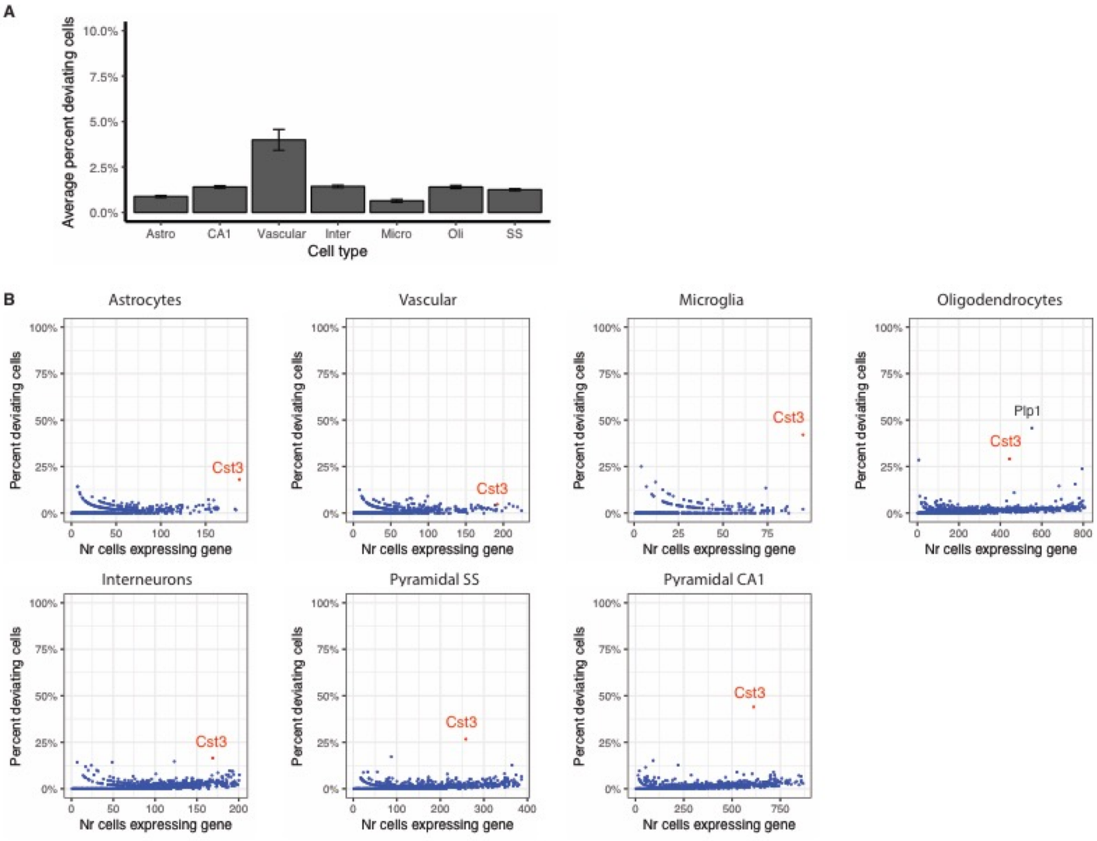

A| Shows average number of cells across genes that deviate from expected ratio of major and minor TSS for all cell types. Error bars show standard error of the mean (SEM)

B| Shows for each gene the number of cells that deviates from expected ratio of major and minor TSS and how it varies with gene expression for all cell types. Each dot is a gene.

Appendix Figure S13: Correlation versus expression for CA1 neurons with 3′ UTR cage peaks

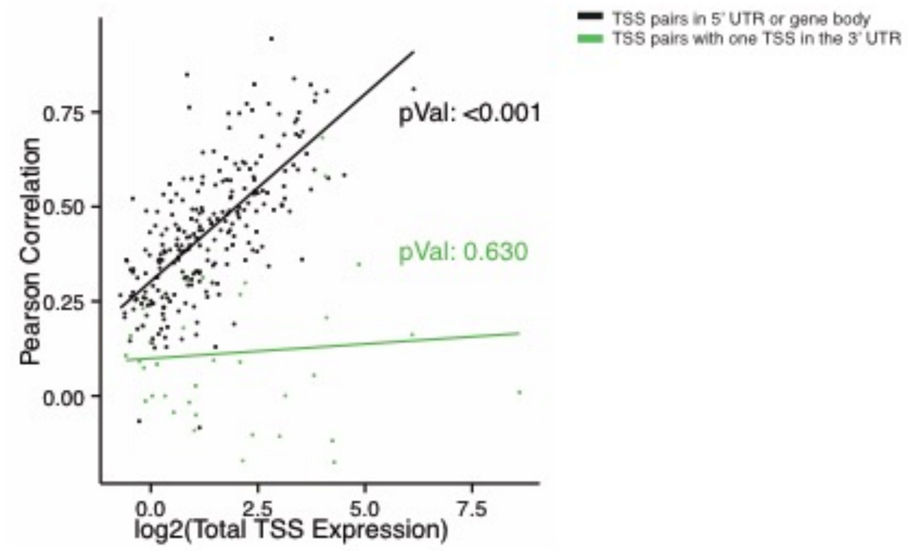

Scatterplot showing total expression (horizontal axis) and major/minor TSS correlation coefficient (vertical). Each dot is a gene. Here cage peaks not associated with a gene were not filtered away allowing for example a CAGE peak to be associated with the 3’ UTR. Coloring shows TSS pairs where both TSSs are in the 5’ UTR or gene body (black) and pairs where either the major or the minor TSS is located in the 3’ UTR (green).

Appendix Figure S14: Distinct peaks of expression within TSSs

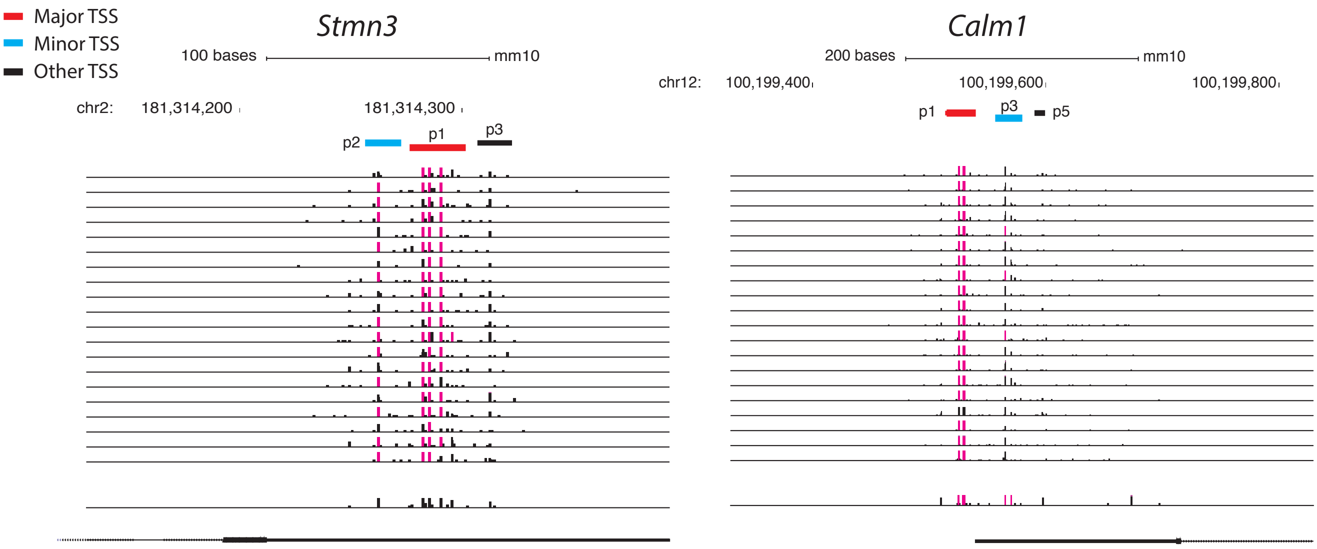

The figure shows two genes with multiple distinct starting positions within the annotated TSS region for CA1 neurons. Expression is shown as bars where the Y-axis for single cells has a limit of 5 molecules and for all cells combined has a limit of 500 molecules. Major TSS is marked in red and minor TSS in blue. Other CAGE defined TSSs and marked in black.

Appendix Figure S15: Examples of genes with two TSS full length gene expression

A. Actin Related Protein 2/3 Complex Subunit 1A (*Arpc1a*)

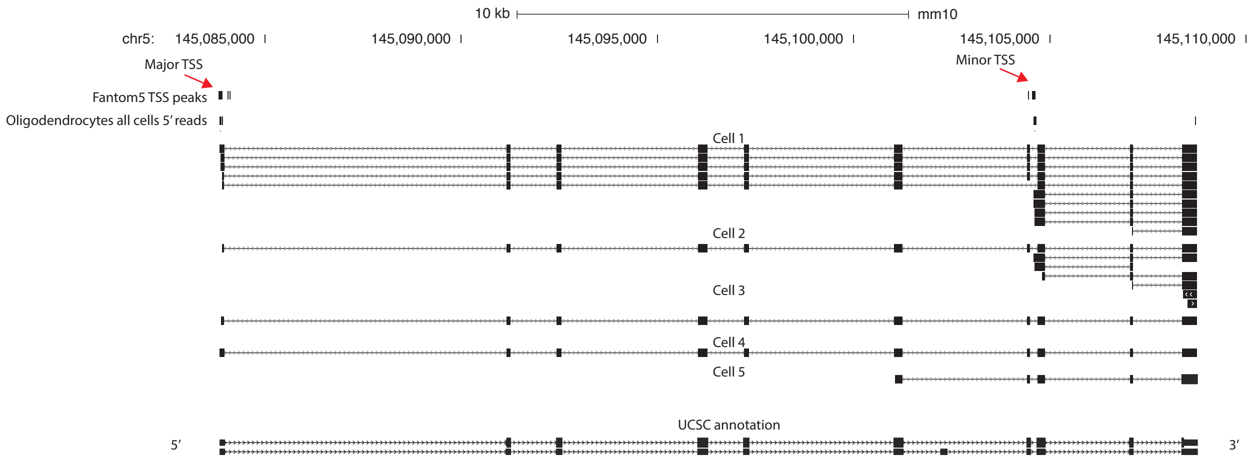

B. 2',3'-Cyclic Nucleotide 3' Phosphodiesterase (*Cnp*)

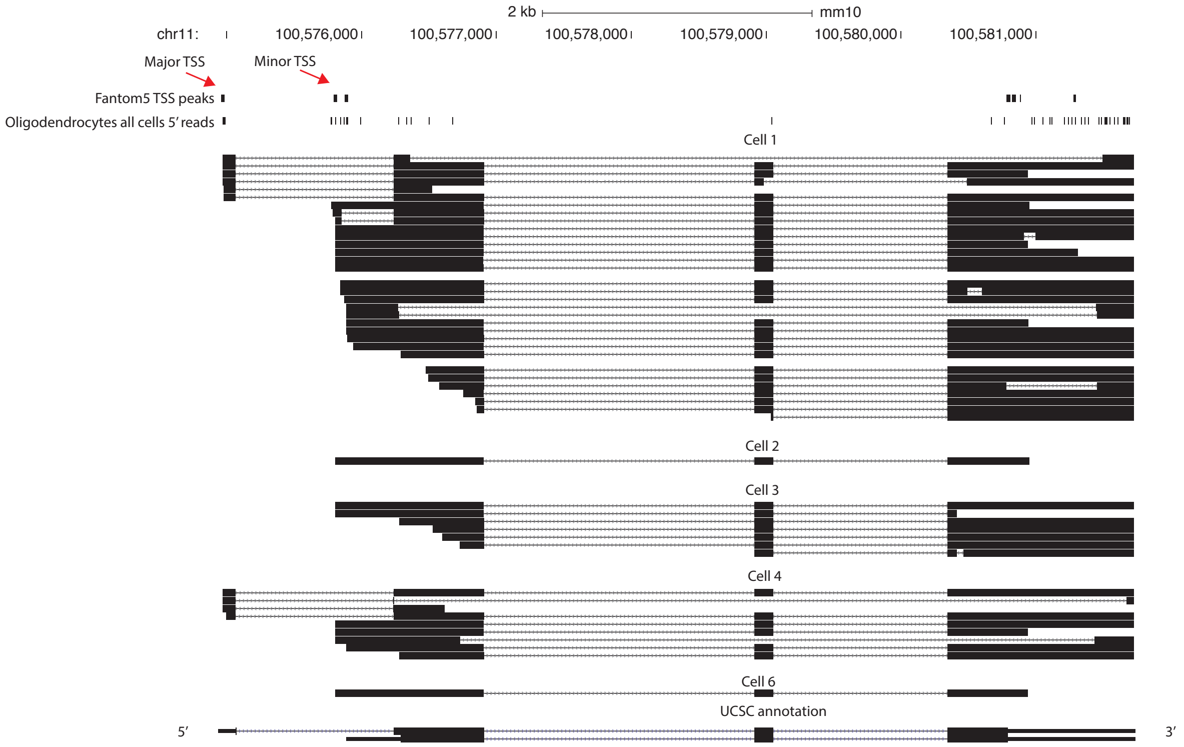

C. Gelsolin (*Gsn*)

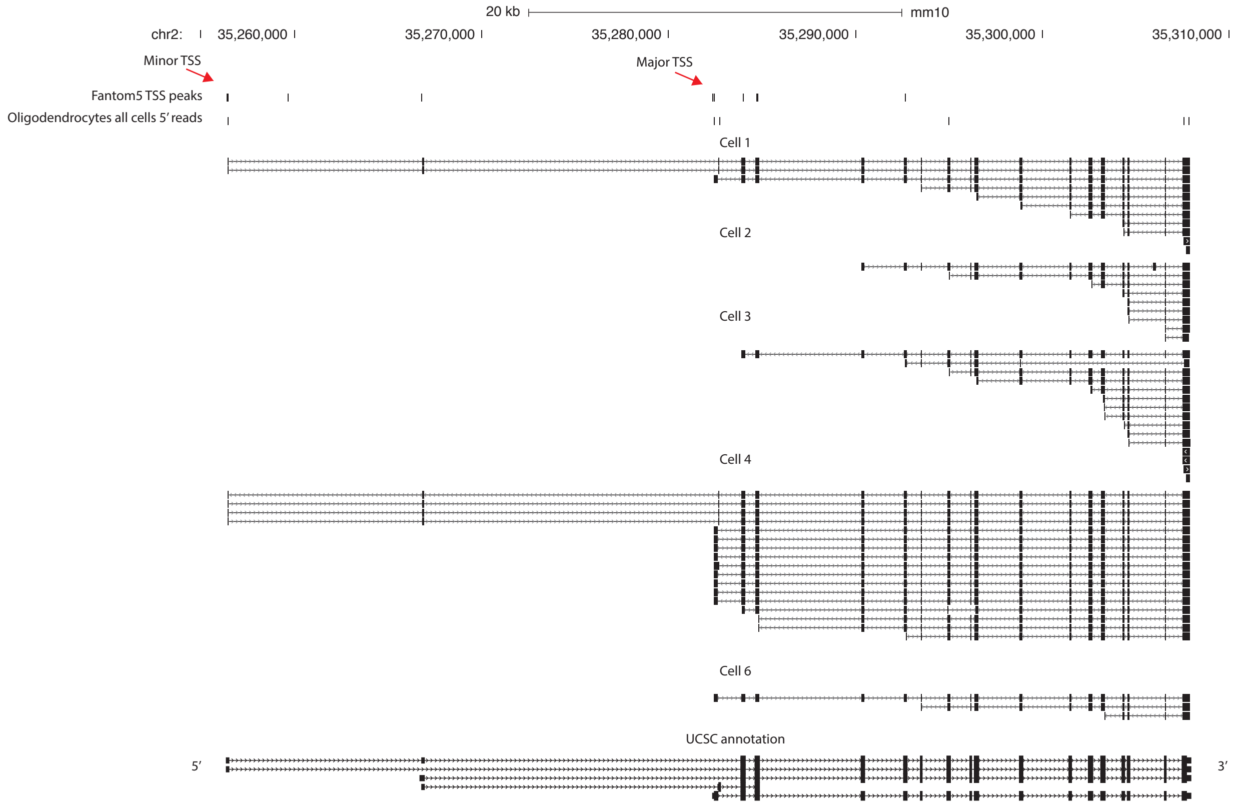

Full length gene expression confirms expression of multiple TSS. The results of PacBio sequencing is shown for 3 genes, Arpc1a, Cnp and Gsn for six individual Oligodendrocyte cells, as well as annotated Fantom 5 TSS peaks and genomic positions with more than 100 molecules from all combined Oligodendrocyte STRT reads. Cells with no expression isn't shown.

Appendix Figure S16 – Cst3 expression across cell types

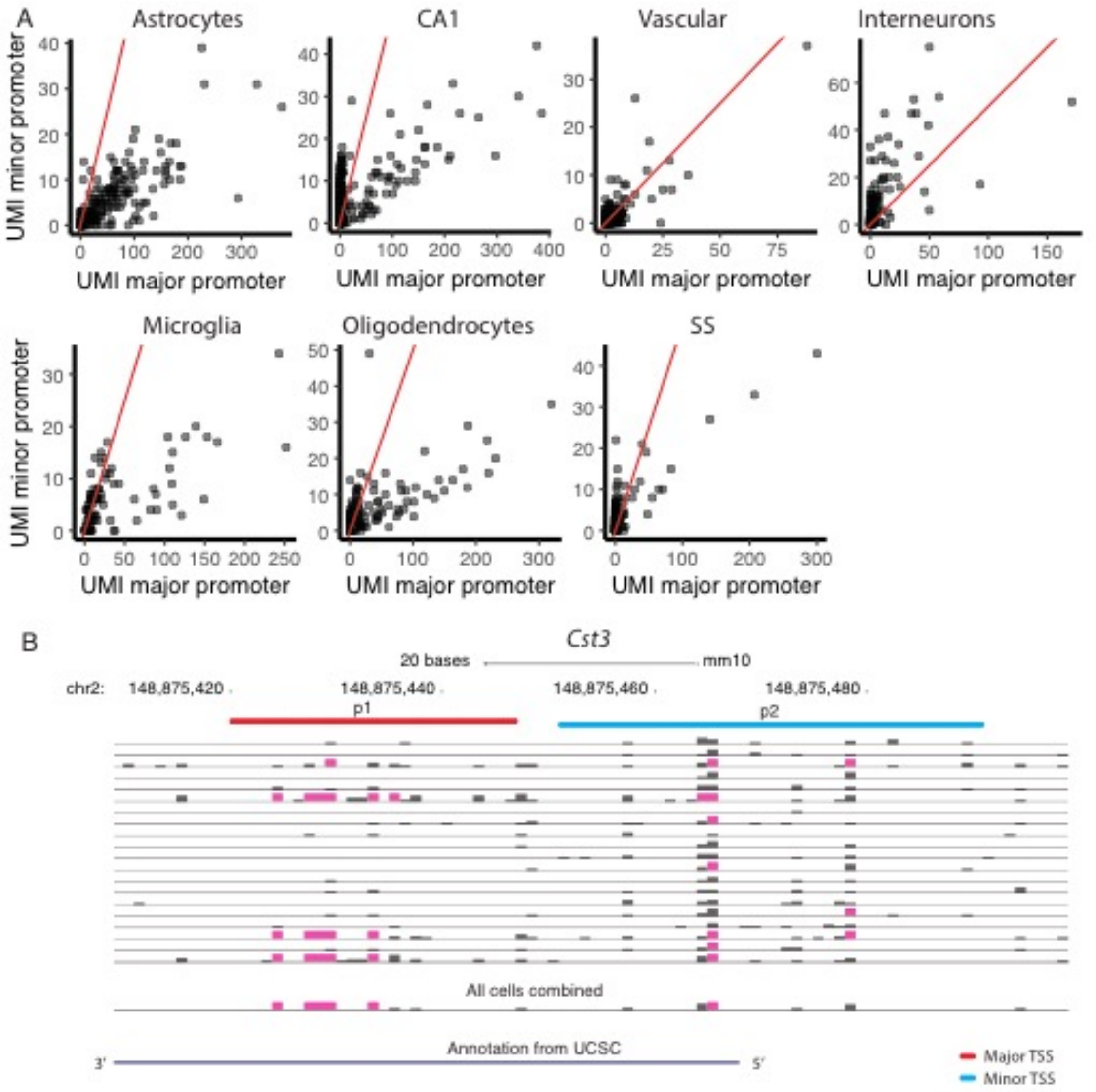

A| TSS expression in single CA1 neuron cells for the *Cst3* gene in various cell types. Plots show the number of mRNA molecules detected from the major and minor TSSs in single cells. Each dot is a single cell. The red line demarks the border between Cst3 high (major TSS highly expressed) and Cst3 low (major TSS lowly expressed), and is the ratio of major/minor TSS of 2:1.

B| Expression of *Cst3* zoomed in on the TSS regions from UCSC browser for CA1 neurons. Expression is shown as bars where the Y-axis for single cells has a limit of 5 molecules and for all cells combined has a limit of 500 molecules.
